# Redox Modulating NRF2: A Potential Mediator of Cancer Stem Cell Resistance

**DOI:** 10.1155/2016/2428153

**Published:** 2015-11-22

**Authors:** In-geun Ryoo, Sang-hwan Lee, Mi-Kyoung Kwak

**Affiliations:** College of Pharmacy, The Catholic University of Korea, Bucheon, Gyeonggi-do 420-743, Republic of Korea

## Abstract

Tumors contain a distinct small subpopulation of cells that possess stem cell-like characteristics. These cells have been called cancer stem cells (CSCs) and are thought to be responsible for anticancer drug resistance and tumor relapse after therapy. Emerging evidence indicates that CSCs share many properties, such as self-renewal and quiescence, with normal stem cells. In particular, CSCs and normal stem cells retain low levels of reactive oxygen species (ROS), which can contribute to stem cell maintenance and resistance to stressful tumor environments. Current literatures demonstrate that the activation of ataxia telangiectasia mutated (ATM) and forkhead box O3 (FoxO3) is associated with the maintenance of low ROS levels in normal stem cells such as hematopoietic stem cells. However, the importance of ROS signaling in CSC biology remains poorly understood. Recent studies demonstrate that nuclear factor-erythroid 2-related factor 2 (NRF2), a master regulator of the cellular antioxidant defense system, is involved in the maintenance of quiescence, survival, and stress resistance of CSCs. Here, we review the recent findings on the roles of NRF2 in maintenance of the redox state and multidrug resistance in CSCs, focusing on how NRF2-mediated ROS modulation influences the growth and resistance of CSCs.

## 1. Introduction

Reactive oxygen species (ROS) are highly proactive molecules derived from molecular oxygen and include free radicals such as hydrogen peroxide (H_2_O_2_), superoxide anion (O_2_^−^), and hydroxyl radical (OH^*∙*^). Under normal physiological conditions, low-to-moderate levels of ROS play a critical role in cellular development and signaling. However, excess ROS levels, which can be caused by metabolic dysfunction or environmental stress conditions, can lead to peroxidation of cellular macromolecules such as lipids, proteins, and nucleic acids [1, 2]. These ROS-induced byproducts eventually trigger cellular senescence, carcinogenesis, or cell death. Interestingly, mammalian cells have developed tightly regulated antioxidant systems for protection against ROS-induced oxidative damage. For example, the superoxide anion, a product of mitochondrial dysfunction, is converted to H_2_O_2_ by superoxide dismutases (SODs). H_2_O_2_ is then decomposed to oxygen and water by catalase or glutathione peroxidases (GPXs) [3, 4].

Multiple lines of evidence suggest that cancer cells possess higher levels of intracellular ROS than normal cells [5, 6]. Elevated ROS levels in cancer can be utilized to promote cell proliferation, invasiveness, and metastasis [6–9]. There are several underlying mechanisms involved in ROS elevation in cancer cells. First, activated oncogenes can trigger ROS production through upregulation of ROS-generating enzymes such as NADPH oxidases (NOXs) [10, 11]. The* RAS* oncogene increases NOX1 expression via the extracellular signal-regulated kinases (ERK) [10] or mitogen-activated protein kinase (MAPK) signaling pathways [11] in human cancers. Overexpression of the* c-MYC* oncogene in normal human fibroblasts induces DNA damage by increasing ROS levels [12]. Mutation of mitochondrial DNA (mtDNA) is a major cause of ROS elevation in cancer cells. Polyak et al. found that seven out of ten colorectal cancer cell lines retained somatic mutations in mtDNA; most of these mutations were detected in mitochondrial genes such as those encoding cytochrome c oxidases 1–3, which has potential implications with respect to increase in mitochondrial ROS [13]. Cancer cells have their own adaptation mechanisms against increased ROS, such as upregulation of ROS scavenging systems. As a result of these systems, malignant transformed cells can utilize ROS as a signal for tumor progression and metastasis [5, 14].

Recent studies are expanding our knowledge about the biological implications of ROS in cancer stem cells (CSCs), which are small subpopulation of cancer cells responsible for tumorigenesis and tumor progression and relapse. Based on increasing evidence for the role of ROS in stem cell biology, lower levels of cellular ROS are considered beneficial for the maintenance of quiescence and chemo/radioresistance of CSCs [15]. In this review, we show current findings illustrating the relationship between ROS and CSC biology and present emerging evidence that nuclear factor-erythroid 2- (NF-E2-) related factor 2 (NRF2) may play a role in CSC growth and resistance.

## 2. CSCs and Resistance to Environmental Stress and Chemotherapy

Tumors contain a small population of cells with stem cell properties, namely, CSCs or tumor-initiating cells (TICs) [16, 17]. These cells are known to play a crucial role in tumor maintenance and relapse. In the 1990s, the first experimental evidence of CSCs was introduced by Bonnet and Dick [18]. In acute myeloid leukemia (AML), it appeared that 0.1 to 1% of the total cell population had tumor-initiating activity. This subpopulation exhibited a CD34^+^/CD38^−^ phenotype and was capable of tumor reconstitution after transplantation into nonobese diabetic/severe combined immune-deficient (NOD/SCID) mice [18]. Since then, multiple lines of evidence have revealed that the CSC population exists in different types of solid tumors, including brain, breast, and colon cancers [19–21].

CSCs are characterized by their self-renewal and differentiation capacity, similar to normal stem cells [16]. Markers of embryonic stem cells (ESCs) such as octamer-binding transcription factor 4 (OCT4), Nanog homeobox (NANOG), and SRY (sex determining region Y)-box 2 (SOX2) are expressed in CSCs, and the Wnt/*β*-catenin, Hedgehog, and Notch pathways are implicated in the self-renewal of CSCs [22–26]. Several CSC-specific surface markers have been identified for the detection and isolation of CSCs from the tumor mass. CD44^+^/CD24^−^ phenotypic cells were isolated from breast cancer tissues and breast carcinoma cell lines and were shown to exhibit self-renewal and high tumorigenic capacity [27]. The CD133^+^ subpopulation from brain tumors demonstrated stem cell properties and showed tumor-initiating capability in NOD/SCID mouse brains [20].

CSCs are considered to be one of the main causes of tumor recurrence after therapy. CSC resistance to conventional anticancer drug therapies and radiotherapy is attributed to increased expression of ROS scavenging molecules, drug transporters, and enhanced DNA repair capacity [28–30]. It has been reported that CSCs contain low levels of endogenous ROS compared to those seen in non-CSCs [31, 32]. In primary AMLs, a subpopulation of low ROS-producing cells demonstrated characteristics of CSCs including quiescence and a CD34^+^/CD38^−^ phenotype [31]. Moreover, Chang et al. observed that this population of low ROS-producing cells exhibited increased expression of stem cell markers (*OCT4* and* NANOG*) and higher chemoresistance and tumorigenicity than the population of high ROS-producing cells in head and neck cancer [32].

The ATP-binding cassette transporter (ABC transporter) family is known to induce multidrug resistance by actively transporting intracellular drugs to outside of the cell [33, 34]. P-glycoprotein (P-gp), multidrug resistance-associated proteins (MRPs), and breast cancer associated protein (BCRP) belong to the ABC transporter family. Since many types of anticancer agents are substrates of these ABC transporters, enhanced expression of P-gp, MRPs, and BCRP is strongly associated with the chemoresistant phenotype of CSCs. Based on this, ABC transporters are often used as a CSC surface marker [28, 35]. The side population (SP), which is a fraction of cells that expresses a high level of BCRP, can be isolated from cancer cells using fluorogenic dye Hoechst 33342. As Hoechst 33342 dye is a substrate of BCRP, the BCRP overexpressing cells exclude this fluorescent dye and thereby a fraction of cells with low fluorescence can be isolated from non-SP cells. This method is now widely used to isolate CSCs from cancer cell lines and specimens using a flow cytometry [36].

## 3. Role of ROS in Stem Cells

Stem cells can be broadly classified into two categories: adult stem cells (e.g., haematopoietic stem cells (HSCs) and neural stem cells) and ESCs. Under homeostatic conditions, these stem cells, particularly adult stem cells, are generally maintained in a quiescent state. However, stem cells are able to escape quiescence and enter the cell cycle for proliferation when they are exposed to metabolic changes [37–40].

ROS are considered as important signaling molecules in stem cell biology. They play a key role in stem cell maintenance by preserving quiescence and protecting against environmental stress [37, 38]. Recent stem cell studies have demonstrated that stem cells contain low levels of intracellular ROS, and this redox status was found to be critical for regulation of stem cell quiescence and self-renewal. Murine ESCs exhibited low levels of intracellular ROS compared to differentiated murine cells, due to the increased level of GSH and thioredoxin (TXN) system [41]. Furthermore, HSCs containing low levels of ROS (i.e., cells with low fluorescent activity following the incubation with 2′,7′-dichlorodihydrofluorescein diacetate (DCFDA), a cell-permeable ROS sensing fluorogenic dye) were highly quiescent and expressed relatively high levels of NOTCH1 and BCRP compared to high DCFDA fluorescent cells. High levels of ROS are cytotoxic, since ROS accumulation in HSCs can lead to cellular prematurity and senescence [42, 43].

ROS have been reported to be involved in stem cell differentiation. Bone marrow mesenchymal stem cells (MSCs) are found in the bone marrow together with HSCs and have the potential to differentiate to adipocytes, osteocytes, and chondrocytes. It was shown that human MSCs are highly resistant to ROS. This phenomenon was linked to low levels of cellular ROS and high levels of SODs, catalase, GPX1, and GSH in MSCs [38, 40]. Elevated antioxidant molecules appear to play a crucial role in the protection of stem cells against oxidative stress. However, under certain circumstances, NOX-derived ROS are associated with MSC differentiation. The treatment of MSCs with antioxidants or interfering RNA of NOX4 prevented adipocyte differentiation of MSCs via cAMP response element-binding protein (CREB) inhibition. Similarly, ESC differentiation to the cardiac lineage was dependent on NOX4-derived ROS [44]. These findings indicate that ROS are important for stem cell fate determination for quiescence or differentiation.

## 4. Redox Signaling Molecules in Stem Cells

**Figure 1:**
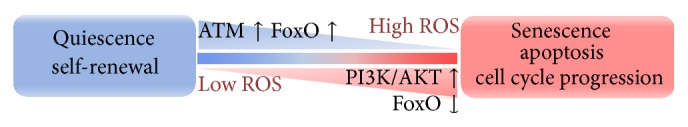
Involvement of ROS in normal stem cell quiescence and self-renewal. In normal stem cells, modulation of ROS levels can determine quiescence and cell fate progression. At low ROS levels, which are maintained by ATM and FoxO signaling, stem cells remain quiescent and self-renewal activity is enhanced. On the other hand, increased ROS levels result in cell cycle progression, cellular senescence, and apoptosis. The PI3K-AKT pathway is known to elevate ROS levels by negative regulation of FoxO.

It has been reported that multiple signaling molecules are involved in ROS-mediated regulation of stem cells (Figure 1). First, ataxia telangiectasia mutated (ATM) plays a critical role in controlling ROS levels in stem cells. ATM, a serine/threonine protein kinase, is a known regulator of the DNA damage response and contributes to the regulation of cellular ROS. ATM is known to regulate ROS via modulation of AMPK-mTOR pathway or NADPH production [45, 46]. Ito et al. showed that* atm* ^−/−^ mice developed bone marrow failure after 24 weeks of age due to a depletion of HSCs. In this study, HSCs in* atm* knockout mice showed higher levels of ROS than wild type mice, which presumably caused a reduction in the self-renewal activity of HSCs. However, the treatment of mice with antioxidant* N*-acetylcysteine (NAC) restored HSC reconstitution in* atm* knockout mice by reducing ROS in HSCs, confirming the critical role of ROS in HSCs maintenance [47]. Similarly, in another study, NAC treatment prevented hypersensitivity of* atm* ^−/−^ mice to X-ray irradiation and senescence of* atm* ^−/−^ embryonic fibroblasts [48]. Cosentino et al. have presented a molecular mechanistic role for ATM, demonstrating that ATM activation promotes the binding of heat shock protein 27 (HSP27) to glucose-6-phosphate dehydrogenase (G6PDH), which can result in G6PDH activation and subsequent NADPH increase [46].

The forkhead box O (FoxO) transcription factor family is also implicated in redox regulation of stem cells. The FoxO family, including FoxO1, FoxO3, FoxO4, and FoxO6, is a key regulator of cell survival, proliferation, DNA repair, and apoptosis. FoxO1 and FoxO3 are reported to upregulate the expression of GSH biosynthetic enzymes and SODs and therefore are associated with cellular protection against oxidative stress [49, 50]. Particularly, FoxO3 has been known to play crucial roles in cytoprotection of stem cells including HSCs [51–53]. Loss of FoxO3a, which regulates the expression of antioxidant enzymes such as catalases and SOD2, led to ROS accumulation and thus a higher rate of cell cycling and a loss of quiescence in HSCs [51]. In* foxo1*/*foxo3a*/*foxo4* triple-knockout mice, the number of HSCs was substantially decreased and apoptotic HSCs were increased through ROS elevation [52]. Notably, Yalcin et al. provided a link between ATM and the FoxO protein in ROS regulation of stem cells. In* foxo3* ^−*/*−^ HSCs, ATM expression was diminished compared to normal HSCs, suggesting that FoxO3 repressed ROS via ATM regulation [53]. Similar to HSCs,* foxo*-deficient neural stem cells demonstrated a decline in self-renewal capacity due to increased cellular ROS levels [54, 55].

The phosphoinositide 3-kinase (PI3K)/AKT pathway is another ROS regulator in normal stem cells. In particular, PI3K/AKT signaling associates with FoxO transcription factors to mediate ROS regulation. Activated AKT promotes FoxO phosphorylation, resulting in the nuclear export and cytoplasmic degradation of FoxO through the proteasome [56, 57]. Therefore,* akt1/2* double knockout HSCs displayed increased quiescence and low cellular ROS levels [58]. Consistently, persistent activation of the PI3K/AKT pathway in phosphatase and tensin homolog (PTEN) deleted HSCs led to defective quiescence, resulting in cellular senescence [59]. Based on the above observations, the PI3K/AKT pathway and FoxO/ATM pathway exhibit opposite roles in ROS regulation of stem cells.

Hypoxia-inducible factors (HIFs) are transcription factors that respond to hypoxic conditions [60]. They are also critical factors for the maintenance of stem cells. HSCs cultured in hypoxic conditions displayed a higher colony formation capacity, and high HIF levels positively regulated the pluripotency of human ESCs by activating stemness transcription factors such as OCT4, SOX2, and NANOG [61, 62]. Moreover, in neuronal stem cells in a hypoxic environment, accumulated HIF1*α* promoted Wnt/*β*-catenin pathway activation [63]. The involvement of HIFs in stem cell biology is mediated by ROS. Takubo et al. observed that* hif1α* ^−/−^ HSCs contain high levels of ROS, which could be associated with a loss of HSC quiescence and an induction of cellular senescence [64]. In agreement with this finding, the suppression of HIF1*α* and HIF2*α* in HSCs led to increased ROS generation via mitochondrial metabolic shift and consequently induced cellular senescence and apoptosis. Scavenging ROS by NAC treatment could restore HSC quiescence and function in stem cells with HIF1*α* and HIF2*α* suppression [65].

## 5. Involvement of ROS in CSC Biology

Very few studies have investigated the involvement of redox change in CSC biology compared to that in cancer cells or normal stem cells. However, it has been reported that CSCs appear to share several ROS-associated properties with normal stem cells. CSCs are known to contain lower levels of ROS compared to non-CSCs. CD44^+^/CD24^−/low^ breast cancer CSCs, isolated from MDA-MB-231 and MCF7 mammospheres, were relatively more resistant to radiation and this was associated with lower levels of ROS after radiation [66]. Diehn et al. observed that ROS levels in CD44^+^/CD24^−^ breast cancer CSCs were lower than in non-CSCs, and the expression levels of the modulatory subunit of glutamate cysteine ligase (GCLM, the rate-limiting enzyme of GSH synthesis) and FoxO1 were high. Treatment of the CD44^+^/CD24^−^ subpopulation with buthionine sulphoximine (BSO), an inhibitor of GSH synthesis, resulted in reduced colony forming capacity and increased sensitivity to radiation therapy through an increase in ROS [67]. In a study using human AML specimens, leukemic stem cells with low levels of ROS majorly contributed to stem cell quiescence by maintaining a low rate of oxidative phosphorylation and metabolism [31]. A leukemia with a high amount of leukemic stem cells showed low levels of ROS and increased expression of GPX3 compared to tumors with a low frequency of leukemic stem cells. This study demonstrated that GPX3 levels positively correlated with poor prognostic outcome in AML patients [68]. Glioma stem cells within the tumor mass have low levels of cellular ROS, although they are located in a hypoxic environment. A proposed molecular mechanism of this phenomenon was the significantly upregulated expression of peroxiredoxin 4 (PRDX4) in glioma stem cells [69].

Evidence is indicating that low ROS levels in CSCs result from the intrinsic characteristics of CSCs. Cell surface markers of CSCs, including CD44 and CD13, are found to be involved in ROS regulation. Ishimoto et al. demonstrated that a variant isoform of CD44 (CD44v) can bind to the cystine/glutamate exchange transporter xCT and activates cysteine uptake to enhance GSH synthesis in gastrointestinal CSCs [70]. The expression of antioxidant genes such as GPX1/2 was significantly increased in CD44^+^ gastric tumor cells. In addition, knockdown of CD44 in mice led to ROS increase, p38MAPK activation, and cellular senescence that are related to p21 expression. In a subsequent study, the same group demonstrated that the number of CD44^+^ cells increased with neoadjuvant chemotherapy in head and neck squamous cell carcinoma (HNSCC) patients. These CD44^+^ undifferentiated cancer cells displayed high xCT expression, GSH upregulation, and low cellular ROS levels. Ablation of xCT by siRNA or sulfasalazine treatment (xCT-mediated cystine transport inhibitor) induced differentiation of HNSCC CSCs both* in vitro* and* in vivo* [71]. CD13 has been identified as a surface marker for liver CSCs. In liver cancer cell lines including Huh7 and PLC/PRF/5, CD13 positive cells predominated the SP fraction and were mainly in the G0/G1 phase of the cell cycle. Additionally, resistance to anticancer drugs or radiation in the CD13 positive cell fraction was much higher than that observed in the CD13 negative cell fraction. Direct comparison of ROS levels between the two cell fractions revealed that the CD13 positive cell fraction contains lower levels of ROS and expresses higher levels of GCLM [72]. In another study by the same group, CD13 expression reduced transforming growth factor-*β* (TGF-*β*) induced ROS production and promoted survival of liver CSCs [73].

It has been demonstrated that signaling pathways involved in ROS regulation of normal stem cells also play a role in CSC biology. The nuclear expression levels of FoxO3a was high in chronic myeloid leukemia-initiating cells, and the transplantation of leukemic stem cells derived from* foxo3a* knockout mice significantly reduced their ability to cause myeloid leukemia in an animal model [74]. This study also revealed that TGF-*β* is a crucial regulator of FoxO3a activity. In the SP of MCF-7 breast cancer cells, activation of the PI3K/mammalian target rapamycin (mTOR) signaling pathway was important for tumorigenecity of these CSCs, and knockdown of PI3K or mTOR led to ablated tumorigenecity [75]. When CD133^+^/CD44^+^ prostate cancer cells were grown in sphere-forming conditions, activated PI3K/AKT signaling was found to be critical for maintaining CSCs [76]. CD44^+^/CD24^−or  low^ cells isolated from breast cancer cell lines and breast cancer patient specimens were radioresistant, and this resistant phenotype was associated with ATM signaling activation [77].

## 6. NRF2 as a Key Molecule for Redox Homeostasis

In 1990, Rushmore and Pickett discovered the enhancer sequence in the rat* gsta2* gene promoter as a response element to *β*-naphthoflavone and* t-*butylhydroquinone (*t*-BHQ) and named it antioxidant responsive element (ARE) [78]. Subsequent studies revealed that ARE is commonly involved in the transcription of multiple antioxidant and detoxifying genes, including glutamate-cysteine ligase (GCL), glutathione* S*-transferase (GST), and NAD(P)H quinone oxidoreductase-1 (NQO-1) [79, 80]. Based on sequence homology between ARE and MAF-recognition element (MRE), further studies hypothesized that small MAF and bZIP cap'n'collar (CNC) transcription factors may interact with ARE [81, 82]. Among bZIP CNC transcription factors, NRF2 was found to play a crucial role in ARE regulation, in which inducible expression of NQO1 and GST was ablated in* t*-butylhydroxy anisole-treated* nrf2* null mice, in contrast to the observation in wild type mice [83]. After this report, numerous studies have elucidated a wide spectrum of protective effects of NRF2 signaling against various stressors. For example, sensitivity to benzo[*a*]pyrene-induced carcinogenesis was significantly greater in* nrf2*-knockout mice than in wild type mice [84].

To account for the protective effects of Nrf2, comparative analyses of gene expression patterns were carried out in* nrf2*-deficient and wild type mice following treatment with Nrf2 activators. In global gene analysis of dithiolethione-administered mouse livers, Nrf2 was found to govern the expression of xenobiotic-detoxifying enzymes, GSH-generating systems, antioxidant proteins, and the molecular chaperone-26S proteasome [85]. Similarly, Hu et al. demonstrated that detoxifying enzymes, antioxidants, drug transporters, stress response proteins, and some signaling molecules serve as Nrf2-dependent and isothiocyanate-inducible genes in mouse liver [86]. It has now been firmly established that NRF2 regulates divergent genes to coordinate xenobiotic detoxification and redox homeostasis [85, 87–89]. In its function as a regulator of cellular redox homeostasis, NRF2 elevates the expression of GCL and the cysteine transporter xCT to increase cellular GSH levels. NRF2 also enhances regeneration of reduced GSH by upregulating GPX and GSH reductase (GSR). Expression of thioredoxin 1 (TXN1), thioredoxin reductase 1 (TXNRD1), and peroxiredoxin 1/6, which can reduce oxidized protein thiols, is also under the control of NRF2. In addition, the levels of NADPH, a cofactor of many antioxidant enzymes such as GSR and TXNRD, can be increased by NRF2 (reviewed in Hayes and Dinkova-Kostova [90]). The expression of multiple NADPH generating enzymes such as G6PDH and 6-phosphogluconate dehydrogenase is upregulated by NRF2. Additionally, the role of NRF2 in ABC transporter expression for xenobiotic detoxification is notable. The basal expression level of Mrp1 was relatively lower in* nrf2*-deficient fibroblasts than that in wild type fibroblasts, and the treatment of mice with Nrf2 activating diethyl maleate increased Mrp1 expression in the liver [91]. Levels of MDR1, MRP2/3, and BCRP were elevated following oltipraz treatment in primary human hepatocytes [92]. Sulforaphane (SFN) treatment enhanced the levels of MDR1, BCRP, and MRP2 in the blood-brain barrier of rats [93]. Our recent study showed that genetic activation of* NRF2* via KEAP1 silencing increases the expression of MDR1, MRP2/3, and BCRP in human proximal tubular epithelial cells [94]. As direct molecular evidence, functional AREs have been identified in human* MRP3* [95] and* BCRP* genes [96].

Kelch-like ECH-associated protein 1 (KEAP1), a cysteine-rich actin-binding protein, is the main negative regulator of NRF2 activity [83, 97]. Under quiescent conditions, NRF2 remains inactive by forming a complex with KEAP1 in the cytoplasm. NRF2 is subject to ubiquitination and KEAP1-induced proteasomal degradation through the Cullin 3 (CUL3) based E3 ligase. KEAP1 has three major domains as follows: (i) The BTB domain is associated with KEAP1 homodimerization, (ii) the IVR domain plays a role in regulation of KEAP1 activity, and (iii) the Kelch/DGR domain mediates binding with NRF2 [83, 90, 98–101]. The binding of NRF2 with KEAP1 has been described as the “hinge and latch” model, where one molecule of NRF2 interacts with the Kelch/DGR domains of the KEAP1 dimer through conserved motifs called ETGE (D/N-X-E-T/S-G-E) and DLG (L-X-X-Q-D-X-D-L-G) [102–104]. In these reports, it was shown that the binding affinity of the ETGE motif to KEAP1 is much higher than that of the DLG motif. It has therefore been shown that “latch” binding of the NRF2 DLG motif is easily broken by modifications of KEAP1 cysteine residues by ROS or electrophiles. In turn, disrupted DLG binding of NRF2 to KEAP1 leads to the blockade of ubiquitination and further degradation of NRF2, resulting in nuclear translocation of NRF2. It has been demonstrated that the sulfhydryl groups of multiple cysteine residues of KEAP1 can be directly modified by oxidation/reduction or alkylation. In particular, Cys151, Cys273, and Cys288 were found to be essential for the regulation of NRF2 activity [99, 104–106]. Mutation of the Cys273 or Cys288 residue of KEAP1 ablated its ability to suppress NRF2 activity, leading to accumulation of the NRF2 protein [99, 104].

In addition to KEAP1-mediated stability regulation, NRF2 activity can be modulated at multiple steps. First, it is noticeable that NRF2 activity is regulated at the transcriptional step. Functional AREs were identified in the murine* nrf2* gene promoter and were involved in the autoregulation of NRF2 through transcriptional activation [107]. Moreover, a single nucleotide polymorphism in the ARE-like sequences of the human* NRF2* promoter was associated with increased lung cancer susceptibility [108]. Second, it was shown that NRF2 activity is regulated by posttranslational modifications. Studies indicate that NRF2 activation involves phosphorylation signaling mediated by multiple kinase pathways such as MAPK, protein kinase C (PKC), PI3K, and protein kinase RNA-like endoplasmic reticulum kinase (PERK) [109–111]. Meanwhile, glycogen synthase kinase-3 (GSK-3), a constitutively active serine/threonine kinase, was found to inhibit NRF2 activity [112]. Last, NRF2 activity is increased by several intrinsic proteins such as p21 and p62 [113, 114]. For example, p62, a linker protein of ubiquitinated proteins to autophagy degradation, binds to the KEAP1 protein and interferes with the binding of NRF2 to KEAP1, resulting in NRF2 stabilization.

## 7. Emerging Role of NRF2 in Cancer Biology

Continuous or fatal stimuli such as toxic chemicals and excess ROS disrupt cellular homeostasis, causing macromolecular damage and alterations in cell cycle and growth signaling, which can eventually result in carcinogenesis. The NRF2 pathway deserved significant attention in the area of cancer biology because numerous studies have demonstrated that activation of the NRF2 pathway decreases the sensitivity of cells to carcinogens [115–117]. For instance, the burden of gastric neoplasia caused by benzo[a]pyrene was effectively attenuated by the Nrf2 activator oltipraz in wild type mice, whereas* nrf2* knockout mice did not show any protective effect of oltipraz [84]. Similarly, the incidence of N-butyl-N-(4-hydroxybutyl)nitrosamine (BBN) induced urinary bladder carcinoma was greater in* nrf2* knockout mice than in wild type mice, and oltipraz treatment reduced tumor incidence only in wild type mice [118].

Although it has been firmly confirmed that NRF2 activation can protect cells against a wide range of toxicants and stressors, aberrant activation of NRF2 has been associated with several types of cancers. NRF2 levels were constitutively elevated in cancer cell lines and tumor samples of the lung, breast, esophagus, endometrial cancers, and prostate cancers [119–125]. Molecular mechanisms involved in constitutive NRF2 activation include the following: (i) somatic mutations of* KEAP1* or* NRF2*, (ii) epigenetic silencing of the* KEAP1* gene, (iii) aberrant accumulation of proteins that compete with NRF2 for KEAP1 binding, and (iv) oncogene-mediated overexpression of NRF2 [101, 126]. First, somatic mutations of* KEAP1*-*NRF2* have been reported in an initial study by Padmanabhan et al. [127]. This study identified mutations in the Kelch/DGR domain of* KEAP1* in lung cancer cell lines as well as lung cancer tissue samples and demonstrated that these mutant KEAP1 proteins lost their NRF2 repressive function, which resulted in NRF2 accumulation. Singh et al. also demonstrated that the Kelch/DGR and IVR domains of the* KEAP1* gene contain multiple somatic mutations and these mutations were identified in 19% of tumor specimens from non-small cell lung cancer patients [123]. In gallbladder cancer, 4 of 13 patients harbored* KEAP1* mutations [122]. Shibata et al. reported that* NRF2* somatic mutations were found in 10.7% of primary lung cancer patients and 27.2% of primary head and neck cancer patients [122]. Notably, these mutations were primarily located in the DLG and ETGE motifs, and eventually led to the loss of a proper interaction between the NRF2 protein and KEAP1. Second, CpG island hypermethylation in the* KEAP1* promoter resulted in low KEAP1 expression in lung cancer cell lines and tumor samples [124]. Third, in human hepatocellular carcinoma, p62 positive cellular aggregates were found with a frequency of 25%, and most of these tumors retained higher levels of NRF2 and its target gene expression [128]. Fourth, oncogenes have also been shown to play a role in NRF2 signaling. Oncogenic activation of KRAS (KRAS^G12D^), c-MYC (c-MYC^ERT12^), and BRAF (BRAF^V619E^) elevates the transcript levels of NRF2 and its target gene expression [129].

It is now widely accepted that aberrant activation of NRF2 can enhance cancer cell survival and growth in oxidizing tumor environments, and further promote chemo/radioresistance. Indeed, the prognosis of cancer patients negatively correlated with NRF2 levels in the tumor [122, 130]. The favorable effect of NRF2 overexpression on tumor survival and growth can be attributed to the increase in NRF2 target antioxidant proteins and their counteractive effect on oxidative stress. For instance, GSH, which is a direct target molecule of NRF2, has been shown to be critical for cell proliferation [131, 132]. In addition to its antioxidant contribution, Mitsuishi et al. provided direct evidence, demonstrating that NRF2 alters the cellular metabolism in relation to anabolic pathways to accelerate cell proliferation [133]. Multiple metabolic genes, such as those involved in the pentose phosphate pathway, were upregulated by NRF2 through ARE, and these changes promoted purine synthesis, glutamine metabolism, and NADPH production for enhanced cell proliferation.

Constitutively high levels of NRF2 have been associated with chemoresistance as well as radioresistance. Cancer cells with high NRF2 activity were less sensitive to cytotoxic chemotherapeutics such as cisplatin, doxorubicin, and 5-fluorouracil through facilitated detoxification of anticancer agents and enhanced antioxidant capacity [101]. It is therefore hypothesized that NRF2 inhibition can enhance the chemosensitivity of cancers. NRF2 siRNA could suppress cancer resistance to cisplatin, topoisomerase inhibitors, and 5-fluorouracil [101, 122, 134, 135]. Cancer cells with constitutively high NRF2 were protected against *γ*-radiation induced toxicity. Moreover, siRNA-mediated inhibition of NRF2 in non-small cell lung cancer cell lines substantially enhanced radiosensitivity [136]. Additionally, NRF2 expression was increased during the acquisition of chemoresistance. In our previous study, doxorubicin-selected ovarian cancer cells demonstrated increased expression of NRF2 and its target genes for GSH synthesis, and NRF2 inhibition in this resistant cell line could restore doxorubicin sensitivity [137].

**Figure 2:**
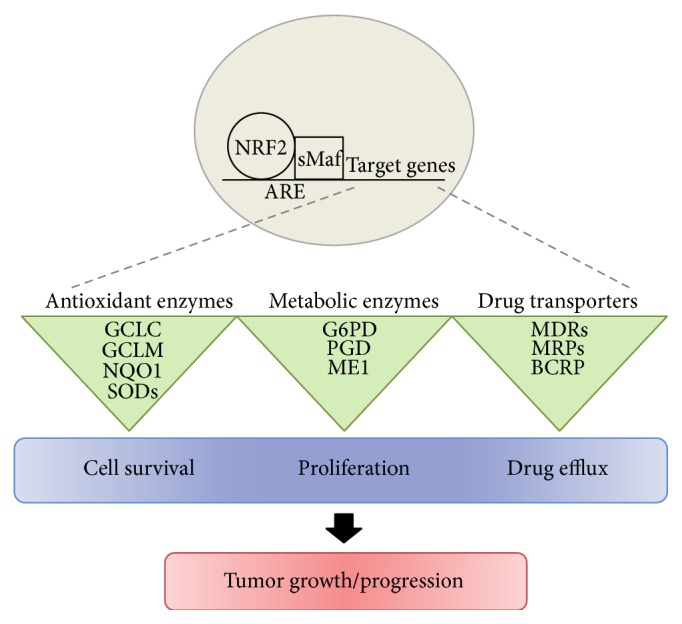
Implications of NRF2 signaling in cancer. NRF2 coordinates the expression of genes associated with cellular redox regulation, metabolism, and xenobiotic efflux, and thereby its aberrant activation promotes cancer cell survival, proliferation, and anticancer drug resistance. GCLM, glutamate cysteine ligase modifier subunit; GCLC, glutamate cysteine ligase catalytic subunit; NQO1, NAD(P)H:quinone oxidoreductase 1; SODs, superoxide dismutases; G6PDH, glucose-6-phosphate dehydrogenase; PGD, phosphogluconate dehydrogenase; ME1, malic enzyme 1, MDRs, multidrug resistance proteins; MRPs, multidrug resistance-associated proteins; BCRP, breast cancer resistance protein.

Our understanding of the role of NRF2 in cancer cell signaling has expanded. In particular, the relationship between oncogenic signaling and NRF2 is noteworthy. As mentioned earlier, activation of oncogenes such as* KRAS* and* c-MYC* increased the expression of NRF2 presumably through oncogene-mediated ROS increase, and this phenomenon appears to contribute to the maintenance of reduced redox homeostasis in cancer cells [129]. In ERBB2 (Her2/Neu) overexpressing ovarian cancer cells, the stable silencing of NRF2 repressed ERBB2 expression and its downstream signaling and retarded tumor growth. Therefore, the inhibition of NRF2 could sensitize these cells to taxol therapy by repressing ERBB2 expression [138]. Moreover, NRF2 was shown to be associated with HIF signaling, which is a critical factor for tumor angiogenesis. When NRF2 was stably knocked down in colon carcinoma cell lines, hypoxia-inducible HIF-1*α* accumulation was abrogated and consequently, angiogenesis and tumor growth were significantly suppressed in* NRF2* knockdown tumors compared to the control group [139]. In type 2 papillary renal cell cancer, which is characterized by loss of the fumarate hydratase gene and consequent metabolic alteration, accumulated fumarate was associated with tumor progression via NRF2 signaling. Fumarate was shown to modify KEAP1 cysteine residues and elevate NRF2 levels, which contributed to the growth and progression of type 2 papillary renal cell cancer [140]. These accumulating lines of evidence suggest that once cells are transformed to the neoplastic stage, cancer cells utilize NRF2 signaling to adapt to the stressful tumor environment and to promote survival and further cancer progression (Figure 2).

## 8. Involvement of NRF2 Signaling in Stem Cell Quiescence and Differentiation

There is considerable evidence to suggest that NRF2 plays a role in normal stem cell biology [141–145]. For example, NRF2 activation in HSCs plays a critical role in not only the maintenance of quiescence but also in the determination of differentiation fate [141, 145]. In* Drosophila* intestinal stem cells, constitutive Nrf2 activation sustained quiescence by reducing the levels of ROS via upregulation of antioxidant genes such as* gclc.* However, in the case of KEAP1-mediated Nrf2 repression, high levels of intracellular ROS facilitated an ablation of the quiescent state in intestinal stem cells and age-related degeneration in the intestinal epithelium [146]. Similarly, low intracellular ROS levels are required for the maintenance of quiescence in human airway basal stem cells (ABSCs). When exposed to exogenous ROS, quiescent ABSCs enter the proliferation stage. Changes in ROS levels activate the NRF2-Notch pathway, which results in self-renewal and protection of ABSCs from ROS-induced hyperproliferation and senescence. Moreover, the quiescent state of ABSCs was maintained by NRF2 activation [147]. In osteoclast progenitor cells, hydrogen sulfide (H_2_S) inhibited human osteoclast differentiation by NRF2-dependent induction of peroxiredoxin 1 and NQO1. These results were further confirmed using NRF2 activators including sulforaphane and* t*-BHQ [148]. It is also notable that NRF2 participates in the regulation of cell fate determination of HSCs. Murakami et al. demonstrated that HSCs derived from* KEAP1*-deficient mice exhibited preferred differentiation into the granulocyte-monocyte lineage rather than differentiating into the erythroid-lymphoid lineage [145].

Up to now, numerous studies have demonstrated that NRF2 plays a protective role against various stressors in stem cells. In neural stem cells, overexpression of NRF2 or pharmacological NRF2 activation prevented necrotic cell death [149]. In an animal study,* nrf2*-deficient mice showed defective stem cell function. HSCs from* nrf2* ^−/−^ mice expressed lower levels of prosurvival cytokines and exhibited spontaneous apoptosis [150]. Ionizing radiation-induced myelosuppression and mortality were mitigated through NRF2-mediated Notch signaling activation in HSCs [142]. Similarly, resveratrol-induced NRF2 expression improved the survival of cardiac stem cells and consequently regenerated infarcted myocardium [151, 152]. The heme oxygenase 1 (HO-1) inducer, cobalt protoporphyrin (CoPP) elicited an antiapoptotic effect on cardiac stem cells via activation of the ERK-NRF2 pathway [153]. In neural stem cells, NRF2 activation by melatonin or* t*-BHQ ameliorated lipopolysaccharide (LPS) or H_2_O_2_ induced cell death [149, 154]. In addition, amyloid *β*-mediated neural stem cell death could be alleviated by exogenous NRF2 transduction, which was accompanied by increased expression of GCLC, NQO-1, and HO-1. This study also demonstrated that neuronal differentiation of neural stem cells is enhanced by NRF2 activation [155]. Similar to neural stem cells, NRF2 has a protective role against hypoxic and oxidative stress conditions in undifferentiated MSCs. Treatment of the murine mesenchymal stem cell line with adrenaline increased the mRNA expression of* nrf2*,* gclc*, and* xCT,* leading to an increase in GSH levels and the prevention of ROS-induced cytotoxicity [156, 157].

## 9. Potential Implication of NRF2 in CSC Maintenance and Resistance

The role of NRF2 in CSC biology is now beginning to be unveiled. Similar to the case of normal stem cells, it was shown that NRF2 contributes to CSC stemness by maintaining their self-renewal capacity and protecting them from chemo/radiotherapy. Achuthan et al. established stable chemotherapy-resistant breast cancer cells and observed that these cells expressed higher levels of CD133 and OCT-4, indicating that these cells exhibit CSC phenotype [34]. Of note, it was shown that ROS levels were relatively low in these drug-selected cells, presumably due to higher levels of antioxidant enzymes such as SOD1 and GPX1/2. NRF2 protein stabilization was associated with high levels of antioxidant enzymes. As an underlying molecular mechanism, diminished proteasome activity and increased p21 levels appear to stabilize the NRF2 protein in these stem-like cells. Evidently, p21 knockdown repressed the mammosphere-forming potential of these stem-like breast cancer cells. Similarly, a study by Zhu et al. showed the involvement of NRF2 in glioblastoma stem cells that were isolated from human surgical glioblastoma specimens.* NRF2* knockdown in glioblastoma stem cells inhibited cell proliferation and neurosphere formation and further suppressed SOX2 expression. Moreover,* NRF2* knockdown changed the cell cycle distribution to the G2 phase and significantly attenuated the tumorigenecity of glioblastoma stem cells [158, 159]. These results are providing evidence that NRF2 is necessary for maintenance of the self-renewal capacity of glioblastoma stem cells.

On the other hand, activation of NRF2 signaling has been demonstrated in different types of CSC models, including lung, esophageal, breast, ovarian, and colon CSCs, and this is closely correlated with the maintenance of low intracellular ROS levels and chemoresistance of CSCs [160–165]. In lung and esophageal cancer cells, cigarette smoke condensate increased the SP as well as BCRP expression, which are hallmarks of CSCs [165]. Promoter analysis revealed that BCRP expression was associated with elevated levels of NRF2, aryl hydrocarbon receptor (AhR), and specificity protein 1 (SP1). Additionally, this study demonstrated that mithramycin diminished BCRP expression via repression of NRF2, AhR, and SP1, and thereby inhibiting the expression of genes associated with CSC-related pathways, resulting in reduced proliferation and tumorigenecity. Similarly, it was reported that lung cancer SP cells exhibited high levels of NRF2 and BCRP expression. These SP cells were highly tumorigenic and possessed self-renewal capacity, compared to non-SP cells [164]. Emmink et al. performed a proteome analysis on collected secretome from highly tumorigenic CSCs and their corresponding nontumorigenic differentiated cells, both of which were established from human colorectal specimens [160]. Subsequent bioinformatic analysis revealed that the CSC secretome contained a large amount of proteins associated with cell survival and protein quality control, compared to differentiated tumor cells. Notably, the CSC secretome contained an NRF2 antioxidant and detoxifying protein signature, in that it included elevated levels of GCLC, GPX2/3, and TXNRD1. This study provided novel evidence that CSCs secrete NRF2 target antioxidant proteins to counteract extracellular stressors and chemotherapeutics. In patients with ovarian clear cell carcinoma, the expression of the CSC maker aldehyde dehydrogenase-1 (ALDH1) was strongly correlated with an advanced clinical stage and reduced progression free survival [161]. Ovarian clear cell carcinoma cells with high ALDH1 expression maintained low levels of ROS compared to ALDH-low cells, and these cells were shown to express higher levels of NRF2 and its target genes.

Two recent studies have demonstrated NRF2 activation in sphere cultures of breast cancer cells, that is one of models of CSCs. Wu et al. showed that mammospheres derived from MCF7 and MDA-MB231 breast cancer cell lines exhibited lower ROS levels compared to their monolayer counterparts. They also showed that levels of NRF2 and target genes such as NQO1 and GCLM were elevated in mammospheres [163]. Similarly, our group has shown substantially elevated NRF2 protein levels along with increased expression of antioxidant genes (e.g.,* HO-1* and* GPX2*) and drug efflux transporters (e.g., MRP2 and BCRP) in sphere cultures of breast cancer cells. NRF2 accumulation was also observed in sphere-cultured ovarian and colon cancer cells. However, shRNA-mediated downregulation of NRF2 led to decreased chemoresistance of mammospheres presumably due to reduced levels of antioxidant genes and drug transporters. High ROS levels in* NRF2* knockdown mammospheres caused sphere growth retardation and apoptosis. Coherently, ablation of ABC transporter induction in* NRF2* knockdown mammospheres sensitized to anticancer agents [162]. Additionally, this study provided evidence that increased NRF2 protein expression in mammospheres can be linked to 26S proteasome reduction and p62 accumulation. In particular, knockdown of* p62* in MCF7 mammospheres significantly attenuated NRF2 elevation.

Surviving dedifferentiated breast cancer cells after chemotherapy treatment retained high levels of NRF2 activation, similar to other CSCs. However, NRF2 activation was mediated by a noncanonical pathway. Levels of PERK were high in dedifferentiated cancer cells and this in turn phosphorylated and activated NRF2 signaling to maintain low cellular ROS levels and to express ABC transporters. In agreement with these findings, clinical observations revealed that the PERK pathway gene signature is related to chemoresistance and reduced patient survival [166].

## 10. Concluding Remarks

**Figure 3:**
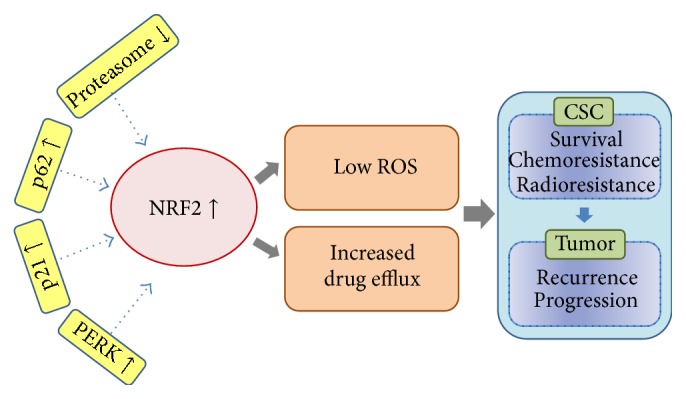
Potential roles of NRF2 signaling in CSCs. In CSC-like cell models, NRF2 is activated through multiple molecular mechanisms in a context-dependent manner. The upregulation of competing proteins such as p62 and p21, activation of PERK, or repressed proteasome function were shown to enhance NRF2 activity in these models. Elevated NRF2 levels in CSCs can contribute to the maintenance of low ROS by upregulating multiple antioxidant genes. In addition, NRF2-mediated expression of ABC transporters elicits efflux of anticancer drugs from cancer cells. Overall, activated NRF2 signaling facilitates CSCs survival and stress resistance, and consequently, it can be suggested that CSCs with high NRF2 activity play a crucial role in tumor recurrence and further progression.

Recent studies have started to uncover the role of ROS signaling in the biology of CSCs, which is related to tumorigenecity, tumor progression, and relapse. Expression of the transcription factor NRF2, a master regulator of antioxidant genes expression, is increased in different models of CSCs, and this elevation is likely to promote CSC maintenance and survival in an oxidizing tumor microenvironment. In addition, NRF2-mediated overexpression of ABC transporters, particularly the CSC marker BCRP, may play a critical role in the multidrug resistance of CSCs. These findings, combined with the increasing evidence showing the alteration of KEAP1-NRF2 signaling in cancer cells, suggest a novel role of NRF2 in CSC maintenance and survival (Figure 3).

One important question that arises from the current studies is whether it is possible to design CSC-targeted therapies through regulation of the NRF2 pathway and its related redox homeostasis in CSCs. Recent studies provide several potential clues for addressing this question: the naturally occurring alkaloid brusatol could reduce the growth and chemoresistance of breast CSCs [163]. Treatment of mammospheres with brusatol elevated ROS levels and promoted taxol-induced growth retardation and cell death. The NRF2 repressive mechanism of brusatol has not been clearly elucidated, but it appears to be independent of KEAP1-mediated degradation [167]. In addition to brusatol, natural compounds such as chrysin, apigenin, luteolin, and trigonelline are known to inhibit NRF2 signaling in several types of cancer cells [102, 168–170] and therefore the development of NRF2 inhibitors with characterized modes of action will enable efficient targeting of the redox homeostasis system as well as multidrug resistance systems in CSCs.

## References

[1] Reczek C. R., Chandel N. S. (2015). ROS-dependent signal transduction. *Current Opinion in Cell Biology*.

[2] Storz P. (2011). Forkhead homeobox type O transcription factors in the responses to oxidative stress. *Antioxidants & Redox Signaling*.

[3] Balaban R. S., Nemoto S., Finkel T. (2005). Mitochondria, oxidants, and aging. *Cell*.

[4] Sena L. A., Chandel N. S. (2012). Physiological roles of mitochondrial reactive oxygen species. *Molecular Cell*.

[5] Schumacker P. T. (2006). Reactive oxygen species in cancer cells: live by the sword, die by the sword. *Cancer Cell*.

[6] Toyokuni S., Okamoto K., Yodoi J., Hiai H. (1995). Persistent oxidative stress in cancer. *FEBS Letters*.

[7] Behrend L., Henderson G., Zwacka R. (2003). Reactive oxygen species in oncogenic transformation. *Biochemical Society Transactions*.

[8] Hileman E. O., Liu J., Albitar M., Keating M. J., Huang P. (2004). Intrinsic oxidative stress in cancer cells: a biochemical basis for therapeutic selectivity. *Cancer Chemotherapy and Pharmacology*.

[9] Pelicano H., Carney D., Huang P. (2004). ROS stress in cancer cells and therapeutic implications. *Drug Resistance Updates*.

[10] Adachi Y., Shibai Y., Mitsushita J., Shang W. H., Hirose K., Kamata T. (2008). Oncogenic Ras upregulates NADPH oxidase 1 gene expression through MEK-ERK-dependent phosphorylation of GATA-6. *Oncogene*.

[11] Mitsushita J., Lambeth J. D., Kamata T. (2004). The superoxide-generating oxidase Nox1 is functionally required for Ras oncogene transformation. *Cancer Research*.

[12] Vafa O., Wade M., Kern S. (2002). c-Myc can induce DNA damage, increase reactive oxygen species, and mitigate p53 function: a mechanism for oncogene-induced genetic instability. *Molecular Cell*.

[13] Polyak K., Li Y., Zhu H. (1998). Somatic mutations of the mitochondrial genome in human colorectal tumours. *Nature Genetics*.

[14] Trachootham D., Alexandre J., Huang P. (2009). Targeting cancer cells by ROS-mediated mechanisms: a radical therapeutic approach?. *Nature Reviews Drug Discovery*.

[15] Shi X., Zhang Y., Zheng J., Pan J. (2012). Reactive oxygen species in cancer stem cells. *Antioxidants & Redox Signaling*.

[16] Reya T., Morrison S. J., Clarke M. F. (2001). Stem cells, cancer, and cancer stem cells. *Nature*.

[17] Zhou B. S., Zhang H., Damelin M., Geles K. G., Grindley J. C., Dirks P. B. (2009). Tumour-initiating cells: challenges and opportunities for anticancer drug discovery. *Nature Reviews Drug Discovery*.

[18] Bonnet D., Dick J. E. (1997). Human acute myeloid leukemia is organized as a hierarchy that originates from a primitive hematopoietic cell. *Nature Medicine*.

[19] O’Brien C. A., Pollett A., Gallinger S., Dick J. E. (2006). A human colon cancer cell capable of initiating tumour growth in immunodeficient mice. *Nature*.

[20] Singh S. K., Hawkins C., Clarke I. D. (2004). Identification of human brain tumour initiating cells. *Nature*.

[21] Al-Hajj M., Wicha M. S., Benito-Hernandez A., Morrison S. J., Clarke M. F. (2003). Prospective identification of tumorigenic breast cancer cells. *Proceedings of the National Academy of Sciences*.

[22] Chiou S.-H., Wang M.-L., Chou Y.-T. (2010). Coexpression of Oct4 and Nanog enhances malignancy in lung adenocarcinoma by inducing cancer stem cell-like properties and epithelial-mesenchymal transdifferentiation. *Cancer Research*.

[23] Yu F., Li J., Chen H. (2011). Kruppel-like factor 4 (KLF4) is required for maintenance of breast cancer stem cells and for cell migration and invasion. *Oncogene*.

[24] Vermeulen L., Felipe De Sousa E. M., van der Heijden M. (2010). Wnt activity defines colon cancer stem cells and is regulated by the microenvironment. *Nature Cell Biology*.

[25] Zhao C., Chen A., Jamieson C. H. (2009). Hedgehog signalling is essential for maintenance of cancer stem cells in myeloid leukaemia. *Nature*.

[26] Farnie G., Clarke R. B. (2007). Mammary stem cells and breast cancer—role of Notch signalling. *Stem Cell Reviews*.

[27] Ponti D. (2005). Isolation and in vitro propagation of tumorigenic breast cancer cells with stem/progenitor cell properties. *Cancer Research*.

[28] Dean M., Fojo T., Bates S. (2005). Tumour stem cells and drug resistance. *Nature Reviews Cancer*.

[29] Blanpain C., Mohrin M., Sotiropoulou P. A., Passegué E. (2011). DNA-damage response in tissue-specific and cancer stem cells. *Cell Stem Cell*.

[30] Dayem A. A., Choi H., Kim J., Cho S. (2010). Role of oxidative stress in stem, cancer, and cancer stem cells. *Cancers*.

[31] Lagadinou E., Sach A., Callahan K. (2013). BCL-2 inhibition targets oxidative phosphorylation and selectively eradicates quiescent human leukemia stem cells. *Cell Stem Cell*.

[32] Chang C., Chen Y., Chou S. (2014). Distinct subpopulations of head and neck cancer cells with different levels of intracellular reactive oxygen species exhibit diverse stemness, proliferation, and chemosensitivity. *Cancer Research*.

[33] Gottesman M. M., Fojo T., Bates S. E. (2002). Multidrug resistance in cancer: role of ATP–dependent transporters. *Nature Reviews Cancer*.

[34] Achuthan S., Santhoshkumar T. R., Prabhakar J., Nair S. A., Pillai M. R. (2011). Drug-induced senescence generates chemoresistant stemlike cells with low reactive oxygen species. *The Journal of Biological Chemistry*.

[35] Zinzi L., Contino M., Cantore M., Capparelli E., Leopoldo M., Colabufo N. A. (2014). ABC transporters in CSCs membranes as a novel target for treating tumor relapse. *Frontiers in Pharmacology*.

[36] Hadnagy A., Gaboury L., Beaulieu R., Balicki D. (2006). SP analysis may be used to identify cancer stem cell populations. *Experimental Cell Research*.

[37] Bigarella C. L., Liang R., Ghaffari S. (2014). Stem cells and the impact of ROS signaling. *Development*.

[38] Chaudhari P., Ye Z., Jang Y. Y. (2014). Roles of reactive oxygen species in the fate of stem cells. *Antioxidants & Redox Signaling*.

[39] Rafalski V. A., Mancini E., Brunet A. (2012). Energy metabolism and energy-sensing pathways in mammalian embryonic and adult stem cell fate. *Journal of Cell Science*.

[40] Wang K., Zhang T., Dong Q., Nice E. C., Huang C., Wei Y. (2013). Redox homeostasis: the linchpin in stem cell self-renewal and differentiation. *Cell Death and Disease*.

[41] Saretzki G., Armstrong L., Leake A., et al (2004). Stress defense in murine embryonic stem cells is superior to that of various differentiated murine cells. *Stem Cells*.

[42] Jang Y. Y., Sharkis S. J. (2007). A low level of reactive oxygen species selects for primitive hematopoietic stem cells that may reside in the low-oxygenic niche. *Blood*.

[43] Ghaffari S. (2008). Oxidative stress in the regulation of normal and neoplastic hematopoiesis. *Antioxidants & Redox Signaling*.

[44] Schmelter M., Ateghang B., Helmig S., Wartenberg M., Sauer H. (2006). Embryonic stem cells utilize reactive oxygen species as transducers of mechanical strain-induced cardiovascular differentiation. *The FASEB Journal*.

[45] Alexander A., Cai S.-L., Kim J. (2010). ATM signals to TSC2 in the cytoplasm to regulate mTORC1 in response to ROS. *Proceedings of the National Academy of Sciences of the United States of America*.

[46] Cosentino C., Grieco D., Costanzo V. (2011). ATM activates the pentose phosphate pathway promoting anti-oxidant defence and DNA repair. *The EMBO Journal*.

[47] Ito K., Hirao A., Arai F. (2004). Regulation of oxidative stress by ATM is required for self-renewal of haematopoietic stem cells. *Nature*.

[48] Ito K., Takubo K., Arai F. (2007). Regulation of reactive oxygen species by Atm is essential for proper response to DNA double-strand breaks in lymphocytes. *The Journal of Immunology*.

[49] Wang Y., Zhou Y., Graves D. T. (2014). FOXO transcription factors: their clinical significance and regulation. *BioMed Research International*.

[50] Lam E., Francis R., Petkovic M. (2006). FOXO transcription factors: key regulators of cell fate. *Biochemical Society Transactions*.

[51] Miyamoto K., Araki K. Y., Naka K. (2007). Foxo3a is essential for maintenance of the hematopoietic stem cell pool. *Cell Stem Cell*.

[52] Tothova Z., Kollipara R., Huntly B. J. (2007). FoxOs are critical mediators of hematopoietic stem cell resistance to physiologic oxidative stress. *Cell*.

[53] Yalcin S., Zhang X., Luciano J. P. (2008). Foxo3 is essential for the regulation of ataxia telangiectasia mutated and oxidative stress-mediated homeostasis of hematopoietic stem cells. *The Journal of Biological Chemistry*.

[54] Paik J., Ding Z., Narurkar R. (2009). FoxOs cooperatively regulate diverse pathways governing neural stem cell homeostasis. *Cell Stem Cell*.

[55] Renault V. M., Rafalski V. A., Morgan A. A. (2009). FoxO3 regulates neural stem cell homeostasis. *Cell Stem Cell*.

[56] Brunet A., Bonni A., Zigmond M. J. (1999). Akt promotes cell survival by phosphorylating and inhibiting a Forkhead transcription factor. *Cell*.

[57] Brunet A., Kanai F., Stehn J., et al (2002). 14-3-3 transits to the nucleus and participates in dynamic nucleocytoplasmic transport. *Journal of Cell Biology*.

[58] Juntilla M. M., Patil V. D., Calamito M., Joshi R. P., Birnbaum M. J., Koretzky G. A. (2010). AKT1 and AKT2 maintain hematopoietic stem cell function by regulating reactive oxygen species. *Blood*.

[59] Yilmaz Ö. H., Valdez R., Theisen B. K. (2006). Pten dependence distinguishes haematopoietic stem cells from leukaemia-initiating cells. *Nature*.

[60] Greer S. N., Metcalf J. L., Wang Y., Ohh M. (2012). The updated biology of hypoxia-inducible factor. *The EMBO Journal*.

[61] Forristal C. E., Wright K. L., Hanley N. A., Oreffo R. O., Houghton F. D. (2009). Hypoxia inducible factors regulate pluripotency and proliferation in human embryonic stem cells cultured at reduced oxygen tensions. *Reproduction*.

[62] Covello K. L., Kehler J., Yu H. (2006). HIF-2*α* regulates Oct-4: effects of hypoxia on stem cell function, embryonic development, and tumor growth. *Genes & Development*.

[63] Mazumdar J., O'Brien W. T., Johnson R. S. (2010). O_2_ regulates stem cells through Wnt/*β*-catenin signalling. *Nature Cell Biology*.

[64] Takubo K., Goda N., Yamada W. (2010). Regulation of the HIF-1alpha level is essential for hematopoietic stem cells. *Cell Stem Cell*.

[65] Kocabas F., Zheng J., Thet S. (2012). Meis1 regulates the metabolic phenotype and oxidant defense of hematopoietic stem cells. *Blood*.

[66] Phillips T. M., McBride W. H., Pajonk F. (2006). The response of CD24^-/low^/CD44^+^ breast cancer-initiating cells to radiation. *JNCI Journal of the National Cancer Institute*.

[67] Diehn M., Cho R. W., Lobo N. A. (2009). Association of reactive oxygen species levels and radioresistance in cancer stem cells. *Nature*.

[68] Herault O., Hope K. J., Deneault E. (2012). A role for GPx3 in activity of normal and leukemia stem cells. *The Journal of Experimental Medicine*.

[69] Kim S., Kwon C., Nakano I. (2014). Detoxification of oxidative stress in glioma stem cells: mechanism, clinical relevance, and therapeutic development. *Journal of Neuroscience Research*.

[70] Ishimoto T., Nagano O., Yae T. (2011). CD44 variant regulates redox status in cancer cells by stabilizing the xCT subunit of system xc(-) and thereby promotes tumor growth. *Cancer Cell*.

[71] Yoshikawa M., Tsuchihashi K., Ishimoto T. (2013). xCT inhibition depletes CD44v-expressing tumor cells that are resistant to EGFR-targeted therapy in head and neck squamous cell carcinoma. *Cancer Research*.

[72] Haraguchi N., Ishii H., Mimori K. (2010). CD13 is a therapeutic target in human liver cancer stem cells. *Journal of Clinical Investigation*.

[73] Kim H. M., Haraguchi N., Ishii H. (2012). Increased CD13 expression reduces reactive oxygen species, promoting survival of liver cancer stem cells via an epithelial-mesenchymal transition-like phenomenon. *Annals of Surgical Oncology*.

[74] Naka K., Hoshii T., Muraguchi T. (2010). TGF-*β*–FOXO signalling maintains leukaemia-initiating cells in chronic myeloid leukaemia. *Nature*.

[75] Zhou J., Wulfkuhle J., Zhang H. (2007). Activation of the PTEN/mTOR/STAT3 pathway in breast cancer stem-like cells is required for viability and maintenance. *Proceedings of the National Academy of Sciences*.

[76] Dubrovska A., Kim S., Salamone R. J. (2009). The role of PTEN/Akt/PI3K signaling in the maintenance and viability of prostate cancer stem-like cell populations. *Proceedings of the National Academy of Sciences of the United States of America*.

[77] Yin H., Glass J. (2011). The phenotypic radiation resistance of CS44^+^/CD24^−or low^ breast cancer cells is mediated through the enhanced activation of ATM signaling. *PLoS ONE*.

[78] Rushmore T. H., Pickett C. (1990). Transcriptional regulation of the rat glutathione S-transferase Ya subunit gene. Characterization of a xenobiotic-responsive element controlling inducible expression by phenolic antioxidants. *The Journal of Biological Chemistry*.

[79] Li Y., Jaiswal A. (1992). Regulation of human NAD (P) H: quinone oxidoreductase gene. Role of AP1 binding site contained within human antioxidant response element. *The Journal of Biological Chemistry*.

[80] Prestera T., Talalay P., Alam J. (1995). Parallel induction of heme oxygenase-1 and chemoprotective phase 2 enzymes by electrophiles and antioxidants: regulation by upstream antioxidant-responsive elements (ARE). *Molecular Medicine*.

[81] Itoh K., Igarashi K., Hayashi N. (1995). Cloning and characterization of a novel erythroid cell-derived CNC family transcription factor heterodimerizing with the small Maf family proteins. *Molecular and Cellular Biology*.

[82] Venugopal R., Jaiswal A. K. (1996). Nrf1 and Nrf2 positively and c-Fos and Fra1 negatively regulate the human antioxidant response element-mediated expression of NAD(P)H:quinone oxidoreductase1 gene. *Proceedings of the National Academy of Sciences*.

[83] Itoh K., Chiba T., Takahashi S. (1997). An Nrf2/small Maf heterodimer mediates the induction of phase II detoxifying enzyme genes through antioxidant response elements. *Biochemical and Biophysical Research Communications*.

[84] Ramos-Gomez M., Kwak M.-K., Dolan P. M. (2001). Sensitivity to carcinogenesis is increased and chemoprotective efficacy of enzyme inducers is lost in nrf2 transcription factor-deficient mice. *Proceedings of the National Academy of Sciences*.

[85] Kwak M.-K., Wakabayashi N., Itoh K., Motohashi H., Yamamoto M., Kensler T. W. (2003). Modulation of gene expression by cancer chemopreventive dithiolethiones through the Keap1-Nrf2 pathway. Identification of novel gene clusters for cell survival. *The Journal of Biological Chemistry*.

[86] Hu R., Xu C., Shen G. (2006). Gene expression profiles induced by cancer chemopreventive isothiocyanate sulforaphane in the liver of C57BL/6J mice and C57BL/6J/Nrf2 (−/−) mice. *Cancer Letters*.

[87] Chanas S., Jiang Q., McMahon M. (2002). Loss of the Nrf2 transcription factor causes a marked reduction in constitutive and inducible expression of the glutathione S-transferase Gsta1, Gsta2, Gstm1, Gstm2, Gstm3 and Gstm4 genes in the livers of male and female mice. *Biochemical Journal*.

[88] Cho H.-Y., Reddy S. P., DeBiase A., Yamamoto M., Kleeberger S. R. (2005). Gene expression profiling of NRF2-mediated protection against oxidative injury. *Free Radical Biology and Medicine*.

[89] Shen G., Xu C., Hu R. (2006). Modulation of nuclear factor E2-related factor 2-mediated gene expression in mice liver and small intestine by cancer chemopreventive agent curcumin. *Molecular Cancer Therapeutics*.

[90] Hayes J. D., Dinkova-Kostova A. T. (2014). The Nrf2 regulatory network provides an interface between redox and intermediary metabolism. *Trends in Biochemical Sciences*.

[91] Hayashi A., Suzuki H., Itoh K., Yamamoto M., Sugiyama Y. (2003). Transcription factor Nrf2 is required for the constitutive and inducible expression of multidrug resistance-associated protein 1 in mouse embryo fibroblasts. *Biochemical and Biophysical Research Communications*.

[92] Jigorel E., Le Vee M., Boursier-Neyret C., Parmentier Y., Fardel O. (2006). Differential regulation of sinusoidal and canalicular hepatic drug transporter expression by xenobiotics activating drug-sensing receptors in primary human hepatocytes. *Drug Metabolism and Disposition*.

[93] Wang X., Campos C. R., Peart J. C. (2014). Nrf2 upregulates ATP binding cassette transporter expression and activity at the blood-brain and blood-spinal cord barriers. *Journal of Neuroscience*.

[94] Jeong H., Ryoo I., Kwak M. (2015). Regulation of the expression of renal drug transporters in KEAP1-knockdown human tubular cells. *Toxicology in Vitro*.

[95] Canet M. J., Merrell M. D., Harder B. G. (2014). Identification of a functional antioxidant response element within the eighth intron of the human ABCC_3_ gene. *Drug Metabolism and Disposition*.

[96] Singh A., Wu H., Zhang P., Happel C., Ma J., Biswal S. (2010). Expression of ABCG2 (BCRP) is regulated by Nrf2 in cancer cells that confers side population and chemoresistance phenotype. *Molecular Cancer Therapeutics*.

[97] Wakabayashi N., Itoh K., Wakabayashi J. (2003). Keap1-null mutation leads to postnatal lethality due to constitutive Nrf2 activation. *Nature Genetics*.

[98] Cullinan S. B., Gordan J. D., Jin J., Harper J. W., Diehl J. A. (2004). The Keap1-BTB protein is an adaptor that bridges Nrf2 to a Cul3-based E3 ligase: oxidative stress sensing by a Cul3-Keap1 ligase. *Molecular and Cellular Biology*.

[99] Zhang D. D., Hannink M. (2003). Distinct cysteine residues in keap1 are required for keap1-dependent ubiquitination of nrf2 and for stabilization of nrf2 by chemopreventive agents and oxidative stress. *Molecular and Cellular Biology*.

[100] Zipper L. M., Mulcahy R. T. (2002). The Keap1 BTB/POZ dimerization function is required to sequester Nrf2 in cytoplasm. *The Journal of Biological Chemistry*.

[101] Jaramillo M. C., Zhang D. D. (2013). The emerging role of the Nrf2-Keap1 signaling pathway in cancer. *Genes & Development*.

[102] Tong K. I., Katoh Y., Kusunoki H., Itoh K., Tanaka T., Yamamoto M. (2006). Keap1 recruits Neh2 through binding to ETGE and DLG motifs: characterization of the two-site molecular recognition model. *Molecular and Cellular Biology*.

[103] Tong K. I., Kobayashi A., Katsuoka F., Yamamoto M. (2006). Two-site substrate recognition model for the Keap1-Nrf2 system: a hinge and latch mechanism. *Biological Chemistry*.

[104] Tong K. I., Padmanabhan B., Kobayashi A. (2007). Different electrostatic potentials define ETGE and DLG motifs as hinge and latch in oxidative stress response. *Molecular and Cellular Biology*.

[105] Kobayashi M., Li L., Iwamoto N. (2009). The antioxidant defense system Keap1-Nrf2 comprises a multiple sensing mechanism for responding to a wide range of chemical compounds. *Molecular and Cellular Biology*.

[106] Yamamoto T., Suzuki T., Kobayashi A. (2008). Physiological significance of reactive cysteine residues of Keap1 in determining Nrf2 activity. *Molecular and Cellular Biology*.

[107] Kwak M. K., Itoh K., Yamamoto M., Kensler T. W. (2002). Enhanced expression of the transcription factor Nrf2 by cancer chemopreventive agents: role of antioxidant response element-like sequences in the nrf2 promoter. *Molecular and Cellular Biology*.

[108] Suzuki T., Shibata T., Takaya K. (2013). Regulatory nexus of synthesis and degradation deciphers cellular Nrf2 expression levels. *Molecular and Cellular Biology*.

[109] Cullinan S. B., Zhang D., Hannink M., Arvisais E., Kaufman R. J., Diehl J. A. (2003). Nrf2 is a direct PERK substrate and effector of PERK-dependent cell survival. *Molecular and Cellular Biology*.

[110] Li W., Kong A. (2009). Molecular mechanisms of Nrf2-mediated antioxidant response. *Molecular Carcinogenesis*.

[111] Surh Y. J. (2003). Cancer chemoprevention with dietary phytochemicals. *Nature Reviews Cancer*.

[112] Salazar M., Rojo A. I., Velasco D., de Sagarra R. M., Cuadrado A. (2006). Glycogen synthase kinase-3beta inhibits the xenobiotic and antioxidant cell response by direct phosphorylation and nuclear exclusion of the transcription factor Nrf2. *Journal of Biological Chemistry*.

[113] Chen W., Sun Z., Wang X. J. (2009). Direct interaction between Nrf2 and p21(Cip1/WAF1) upregulates the Nrf2-mediated antioxidant response. *Molecular Cell*.

[114] Komatsu M., Kurokawa H., Waguri S. (2010). The selective autophagy substrate p62 activates the stress responsive transcription factor Nrf2 through inactivation of Keap1. *Nature Cell Biology*.

[115] Hayes J. D., McMahon M., Chowdhry S., Dinkova-Kostova A. T. (2010). Cancer chemoprevention mechanisms mediated through the Keap1-Nrf2 pathway. *Antioxidants & Redox Signaling*.

[116] Hu R., Saw C. L., Yu R., Kong A. T. (2010). Regulation of NF-E2-related factor 2 signaling for cancer chemoprevention: antioxidant coupled with antiinflammatory. *Antioxidants & Redox Signaling*.

[117] Kwak M., Kensler T. W. (2010). Targeting NRF2 signaling for cancer chemoprevention. *Toxicology and Applied Pharmacology*.

[118] Iida K., Itoh K., Kumagai Y. (2004). Nrf2 is essential for the chemopreventive efficacy of oltipraz against urinary bladder carcinogenesis. *Cancer Research*.

[119] Jiang T., Chen N., Zhao F. (2010). High levels of Nrf2 determine chemoresistance in type II endometrial cancer. *Cancer Research*.

[120] Kim Y. R., Oh J. E., Kim M. S. (2010). Oncogenic *NRF2* mutations in squamous cell carcinomas of oesophagus and skin. *The Journal of Pathology*.

[121] Shibata T., Kokubu A., Gotoh M. (2008). Genetic alteration of Keap1 confers constitutive Nrf2 activation and resistance to chemotherapy in gallbladder cancer. *Gastroenterology*.

[122] Shibata T., Ohta T., Tong K. I. (2008). Cancer related mutations in NRF2 impair its recognition by Keap1-Cul3 E3 ligase and promote malignancy. *Proceedings of the National Academy of Sciences of the United States of America*.

[123] Singh A., Misra V., Thimmulappa R. K. (2006). Dysfunctional KEAP1-NRF2 interaction in non-small-cell lung cancer. *PLoS Medicine*.

[124] Wang R., An J., Ji F., Jiao H., Sun H., Zhou D. (2008). Hypermethylation of the *Keap1* gene in human lung cancer cell lines and lung cancer tissues. *Biochemical and Biophysical Research Communications*.

[125] Zhang P., Singh A., Yegnasubramanian S. (2010). Loss of Kelch-like ECH-associated protein 1 function in prostate cancer cells causes chemoresistance and radioresistance and promotes tumor growth. *Molecular Cancer Therapeutics*.

[126] Hayes J. D., McMahon M. (2009). NRF2 and KEAP1 mutations: permanent activation of an adaptive response in cancer. *Trends in Biochemical Sciences*.

[127] Padmanabhan B., Tong K. I., Ohta T. (2006). Structural basis for defects of Keap1 activity provoked by its point mutations in lung cancer. *Molecular Cell*.

[128] Inami Y., Waguri S., Sakamoto A. (2011). Persistent activation of Nrf2 through p62 in hepatocellular carcinoma cells. *The Journal of Cell Biology*.

[129] DeNicola G. M., Karreth F. A., Humpton T. J. (2011). Oncogene-induced Nrf2 transcription promotes ROS detoxification and tumorigenesis. *Nature*.

[130] Solis L. M., Behrens C., Dong W. (2010). Nrf2 and Keap1 abnormalities in non-small cell lung carcinoma and association with clinicopathologic features. *Clinical Cancer Research*.

[131] Reddy N. M., Kleeberger S. R., Cho H. Y. (2007). Deficiency in Nrf2-GSH signaling impairs type II cell growth and enhances sensitivity to oxidants. *American Journal of Respiratory Cell and Molecular Biology*.

[132] Reddy N. M., Kleeberger S. R., Yamamoto M. (2007). Genetic dissection of the Nrf2-dependent redox signaling-regulated transcriptional programs of cell proliferation and cytoprotection. *Physiological Genomics*.

[133] Mitsuishi Y., Taguchi K., Kawatani Y. (2012). Nrf2 redirects glucose and glutamine into anabolic pathways in metabolic reprogramming. *Cancer Cell*.

[134] Cho J., Manandhar S., Lee H., Park H., Kwak M. (2008). Role of the Nrf2-antioxidant system in cytotoxicity mediated by anticancer cisplatin: implication to cancer cell resistance. *Cancer Letters*.

[135] Ohta T., Iijima K., Miyamoto M. (2008). Loss of Keap1 function activates Nrf2 and provides advantages for lung cancer cell growth. *Cancer Research*.

[136] Singh A., Bodas M., Wakabayashi N., Bunz F., Biswal S. (2010). Gain of Nrf2 function in non-small-cell lung cancer cells confers radioresistance. *Antioxidants & Redox Signaling*.

[137] Shim G.-S., Manandhar S., Shin D.-H., Kim T., Kwak M. (2009). Acquisition of doxorubicin resistance in ovarian carcinoma cells accompanies activation of the NRF2 pathway. *Free Radical Biology and Medicine*.

[138] Manandhar S., Choi B., Jung K. (2012). NRF2 inhibition represses ErbB2 signaling in ovarian carcinoma cells: implications for tumor growth retardation and docetaxel sensitivity. *Free Radical Biology and Medicine*.

[139] Kim T., Hur E.-g., Kang S. J. (2011). NRF2 blockade suppresses colon tumor angiogenesis by inhibiting hypoxia-induced activation of HIF-1*α*. *Cancer Research*.

[140] Kinch L., Grishin N. V., Brugarolas J. (2011). Succination of Keap1 and activation of Nrf2-dependent antioxidant pathways in FH-deficient papillary renal cell carcinoma type 2. *Cancer Cell*.

[141] Tsai J. J., Dudakov J. A., Takahashi K. (2013). Nrf2 regulates haematopoietic stem cell function. *Nature Cell Biology*.

[142] Kim J. H., Thimmulappa R. K., Kumar V. (2014). NRF2-mediated Notch pathway activation enhances hematopoietic reconstitution following myelosuppressive radiation. *The Journal of Clinical Investigation*.

[143] Chute J. P. (2014). NRF2 mitigates radiation-induced hematopoietic death. *The Journal of Clinical Investigation*.

[144] Jang J., Wang Y., Kim H., Lalli M. A., Kosik K. S. (2014). Nrf2, a regulator of the proteasome, controls self-renewal and pluripotency in human embryonic stem cells. *Stem Cells*.

[145] Murakami S., Shimizu R., Romeo P., Yamamoto M., Motohashi H. (2014). Keap1-Nrf2 system regulates cell fate determination of hematopoietic stem cells. *Genes to Cells*.

[146] Hochmuth C. E., Biteau B., Bohmann D., Jasper H. (2011). Redox regulation by Keap1 and Nrf2 controls intestinal stem cell proliferation in *Drosophila*. *Cell Stem Cell*.

[147] Paul M. K., Bisht B., Darmawan D. O. (2014). Dynamic changes in intracellular ROS levels regulate airway basal stem cell homeostasis through Nrf2-dependent notch signaling. *Cell Stem Cell*.

[148] Gambari L., Lisignoli G., Cattini L., Manferdini C., Facchini A., Grassi F. (2014). Sodium hydrosulfide inhibits the differentiation of osteoclast progenitor cells via NRF2-dependent mechanism. *Pharmacological Research*.

[149] Li J., Johnson D., Calkins M., Wright L., Svendsen C., Johnson J. (2005). Stabilization of Nrf2 by tBHQ confers protection against oxidative stress-induced cell death in human neural stem cells. *Toxicological Sciences*.

[150] Merchant A. A., Singh A., Matsui W., Biswal S. (2011). The redox-sensitive transcription factor Nrf2 regulates murine hematopoietic stem cell survival independently of ROS levels. *Blood*.

[151] Gurusamy N., Ray D., Lekli I., Das D. K. (2010). Red wine antioxidant resveratrol-modified cardiac stem cells regenerate infarcted myocardium. *Journal of Cellular and Molecular Medicine*.

[152] Gorbunov N., Petrovski G., Gurusamy N., Ray D., Kim D. H., Das D. K. (2012). Regeneration of infarcted myocardium with resveratrol-modified cardiac stem cells. *Journal of Cellular and Molecular Medicine*.

[153] Cai C., Teng L., Vu D. (2012). The heme oxygenase 1 inducer (CoPP) protects human cardiac stem cells against apoptosis through activation of the extracellular signal-regulated kinase (ERK)/NRF2 signaling pathway and cytokine release. *The Journal of Biological Chemistry*.

[154] Song J., Kang S. M., Lee K. M., Lee J. E. (2015). The protective effect of melatonin on neural stem cell against LPS-induced inflammation. *BioMed Research International*.

[155] Kärkkäinen V., Pomeshchik Y., Savchenko E. (2014). Nrf2 regulates neurogenesis and protects neural progenitor cells against A*β* toxicity. *STEM CELLS*.

[156] Mohammadzadeh M., Halabian R., Gharehbaghian A. (2012). Nrf-2 overexpression in mesenchymal stem cells reduces oxidative stress-induced apoptosis and cytotoxicity. *Cell Stress and Chaperones*.

[157] Takahata Y., Takarada T., Iemata M. (2009). Functional expression of *β*2 adrenergic receptors responsible for protection against oxidative stress through promotion of glutathione synthesis after Nrf2 upregulation in undifferentiated mesenchymal C3H10T1/2 stem cells. *Journal of Cellular Physiology*.

[158] Zhu J., Wang H., Fan Y. (2014). Knockdown of nuclear factor erythroid 2-related factor 2 by lentivirus induces differentiation of glioma stem-like cells. *Oncology Reports*.

[159] Zhu J., Wang H., Sun Q. (2013). Nrf2 is required to maintain the self-renewal of glioma stem cells. *BMC Cancer*.

[160] Emmink B. L., Verheem A., Van Houdt W. J. (2013). The secretome of colon cancer stem cells contains drug-metabolizing enzymes. *Journal of Proteomics*.

[161] Mizuno T., Suzuki N., Makino H. (2015). Cancer stem-like cells of ovarian clear cell carcinoma are enriched in the ALDH-high population associated with an accelerated scavenging system in reactive oxygen species. *Gynecologic Oncology*.

[162] Ryoo I.-G., Choi B.-H., Kwak M.-K. (2015). Activation of NRF2 by p62 and proteasome reduction in sphere-forming breast carcinoma cells. *Oncotarget*.

[163] Wu T., Harder B. G., Wong P. K., Lang J. E., Zhang D. D. (2014). Oxidative stress, mammospheres and Nrf2-new implication for breast cancer therapy?. *Molecular Carcinogenesis*.

[164] Yang B., Ma Y., Liu Y. (2015). Elevated expression of Nrf-2 and ABCG2 involved in multi-drug resistance of lung cancer SP cells. *Drug Research*.

[165] Zhang M., Mathur A., Zhang Y. (2012). Mithramycin represses basal and cigarette smoke-induced expression of abcg2 and inhibits stem cell signaling in lung and esophageal cancer cells. *Cancer Research*.

[166] Del Vecchio C. A., Feng Y., Sokol E. S. (2014). De-differentiation confers multidrug resistance via noncanonical PERK-Nrf2 signaling. *PLoS Biology*.

[167] Ren D., Villeneuve N. F., Jiang T. (2011). Brusatol enhances the efficacy of chemotherapy by inhibiting the Nrf2-mediated defense mechanism. *Proceedings of the National Academy of Sciences of the United States of America*.

[168] Arlt A., Sebens S., Krebs S. (2013). Inhibition of the Nrf2 transcription factor by the alkaloid trigonelline renders pancreatic cancer cells more susceptible to apoptosis through decreased proteasomal gene expression and proteasome activity. *Oncogene*.

[169] Gao A.-M., Ke Z.-P., Shi F., Sun G., Chen H. (2013). Chrysin enhances sensitivity of BEL-7402/ADM cells to doxorubicin by suppressing PI3K/Akt/Nrf2 and ERK/Nrf2 pathway. *Chemico-Biological Interactions*.

[170] Gao A. M., Ke Z. P., Wang J. N., Yang J., Chen S., Chen H. (2013). Apigenin sensitizes doxorubicin-resistant hepatocellular carcinoma BEL-7402/ADM cells to doxorubicin via inhibiting PI3K/Akt/Nrf2 pathway. *Carcinogenesis*.

